# Announcement—Section on Surgery

**Published:** 1898-04

**Authors:** B. E. Hadra, J. R. Stuart

**Affiliations:** Chairman, San Antonio, Texas; Secretary, Houston, Texas


### Announcement—Section on Surgery.

HOUSTON, TEXAS, March 1st, 1898.

*To the Members of the State Medical Association and the Profession of the State at Large:*

DEAR DOCTOR:—The Texas State Medical Association will meet in this city from the 26th to the 29th of April next. The present indications are that it will be a large and successful meeting.

A prominent feature of these annual reunions has always been the proceedings of the Section on Surgery, and we are consequently desirous that the coming session shall not fall one whit behind its predecessors. To this end we most respectfully ask that you contribute a paper, or report of cases upon some subject pertaining to surgery, and send the title of same to the Secretary not later than the 10th of April.

Yours truly,

B. E. HADRA, Chairman, San Antonio.

J. R. STUART, Secretary, Houston.

